# MicroRNA-608 and MicroRNA-34a Regulate Chordoma Malignancy by Targeting EGFR, Bcl-xL and MET

**DOI:** 10.1371/journal.pone.0091546

**Published:** 2014-03-12

**Authors:** Ying Zhang, David Schiff, Deric Park, Roger Abounader

**Affiliations:** 1 Departments of Microbiology, Immunology and Cancer Biology, University of Virginia, Charlottesville, Virginia, United States of America; 2 Department of Neurology, University of Virginia, Charlottesville, Virginia, United States of America; 3 Department of Neurosurgery, University of Virginia, Charlottesville, Virginia, United States of America; 4 Cancer Center, University of Virginia, Charlottesville, Virginia, United States of America; University of Nebraska Medical Center, United States of America

## Abstract

Chordomas are rare malignant tumors that originate from the notochord remnants and occur in the skull base, spine and sacrum. Due to a very limited understanding of the molecular pathogenesis of chordoma, there are no adjuvant and molecular therapies besides surgical resection and radiation therapy. microRNAs (miRNAs) are small noncoding regulatory RNA molecules with critical roles in cancer. The role of miRNAs in chordomas is mostly unknown. We uncover microRNA-608 (miR-608) and microRNA-34a (miR-34a) as novel tumor suppressive microRNAs that regulate malignancy in chordoma. We find that miR-608 and miR-34a expressions are downregulated in human chordoma cell lines and primary cells at least partially via alteration of their genes’ copy numbers. We identify the commonly deregulated oncogenes EGFR and Bcl-xL as direct targets of miR-608 and the receptor tyrosine kinase MET as direct target of miR-34a. We show that EGFR and MET activations promote chordoma cell proliferation and invasion and that pharmacological inhibition of EGFR and MET inhibits chordoma cell proliferation and survival. We demonstrate that restoration of miR-608 and miR-34a inhibits cell proliferation and invasion and induces apoptosis in chordoma cells. We find that miR-34a inversely correlates with MET expression and miR-608 inversely correlates with EGFR expression in chordoma cells. These findings demonstrate for the first time that miR-608 and miR-34a regulate chordoma malignancy by regulating EGFR, MET and Bcl-xL.

## Introduction

Chordomas are rare malignant tumors that develop from persistent notochord tissue. These tumors typically occur in the midline skeleton, most commonly in the skull base and spine. The poor prognosis is mainly due to aggressive local growth, local recurrence and distant metastasis. Current treatments include surgical resection and radiotherapy. There are no drugs that are currently approved to treat chordoma. Despite the most advanced skull base surgical techniques, chordomas are extremely difficult to eradicate by surgery because of the need to preserve adjacent vital structures and recurrence rates are high (40%) [1] [2]. When resection and radiotherapy have been exhausted, patients are left without further therapeutic options. The overall survival time remains at ∼ 5 years [3]. Therefore, there exists significant clinical need for improved therapeutic options for this deadly disease. The development of new therapeutic options is hampered by a very limited knowledge of the molecular basis of chordoma. Among the very few molecular dysregulations that have been associated with chordoma malignancy are the frequent dysregulations of the receptor tyrosine kinases (RTKs), EGFR, PDGFR and MET [4] [5]. However information about the modes of dysregulation of these regulators of chordoma malignancy is lacking. This study uncovers for the first time microRNA dysregulation as an important regulator of RTKs and chordoma malignancy.

microRNAs (miRNAs) are small noncoding regulatory RNA molecules, that have a wide impact on the regulation of gene expression [6]. miRNAs regulate their targets by direct cleavage of the mRNA or by inhibition of protein synthesis, according to the degree of complementarities with their targets’ 3′UTR regions. Many miRNA genes are located at fragile sites in the genome or regions that are commonly amplified or deleted in human cancers [6] [7]. Deregulation of miRNAs that target the expression of oncogenes or tumor suppressor genes can therefore contribute to cancer formation and growth [8], [9].

Very little is known about miRNAs in chordoma. It has been reported that miR-1, miR-31 and potentially miR-663a act as a tumor suppressive miRNAs in chordoma [10]–[13]. We screened human chordoma cell lines and primary cells for miRNA expression by quantitative RT-PCR. We found that miR-608 and miR-34a levels were significantly lower in chordoma cells as compared to normal cells. We therefore investigated the functions and targets of miR-608 and miR-34a in chordoma. Our data show that miR-608 targets and downregulates the receptor tyrosine kinase (RTK) EGFR and the apoptosis inhibitor Bcl-xL, and that miR-34a targets and downregulates the RTK MET. Overexpression of these two microRNAs inhibited chodoma cell proliferation and invasion and induced apoptosis. Thus, loss of miR-608 or miR-34a could enhance chordoma malignancy by inducing overexpression of EGFR, MET and inhibiting apoptosis. The findings propose miR-608 and miR-34a as new tumor suppressors and potential therapeutic agents in chordoma and shed new light onto the very little understood molecular mechanisms of chordoma malignancy.

## Materials and Methods

### Cells, tumor specimens, tissue culture, and reagents

Human chordoma cell lines, UCH1 and UCH2, chordoma primary cells C22, C24, C25, C28, and human fibroblast and astrocyte cells were used for this study. UCH1 [14] and UCH2 [15] were obtained from the Chordoma Foundation and were grown in a 4:1 mixture of DMEM-F12 medium and RPMI-1640 medium containing 10% fetal bovine serum (FBS), 1 mM L-glutamine, and 26 mM sodium bicarbonate in a humidified incubator containing 5% O_2_ and CO_2_ at 37°C. Normal human astrocytes were purchased from Lonza (Basel, Switzerland) and grown in basal medium supplemented with growth factors according to the vendor’s instructions. Tumor specimens were surgically resected from patients with clival chordoma at the University of Pittsburgh Medical Center. All tissues included in the study were confirmed to be chordoma by the clinical pathologist. Primary cultures were established from acutely resected surgical materials. Tumor tissue was placed into sterile DMEM-F12 tissue culture medium in the operating room and immediately transported to the laboratory on ice where they were minced with a scalpel and mechanically dissociated by trituration. Primary cells were passaged upon achieving confluence and used up to 10 passages as long as they retained the physaliphorous morphology. Fibroblasts were purchased from ATCC and grown in DMEM media with 10% of FBS in 37°C in 5% CO_2_, 95% air in the presence of penicillin and streptomycin. Fibroblasts were used as controls because of the near impossibility of obtaining and culturing human notochord tissue. Chordoma surgical specimens which were used from primary cell isolation as well as for whole tissue analysis were obtained with approval of the Institutional Review Board of the University of Pittsburgh (Pittsburgh, PA) after written patient consent. All tissues were confirmed as chordomas by experienced pathologists.

DMEM-F12, DMEM and RPMI were purchased from Invitrogen (La Jolla, CA). Penicillin-streptomycin and Oligofectamine were purchased from Invitrogen (Carlsbad, CA). FBS was purchased from Gemini BioProducts (West Sacramento, CA). Human recombinant EGF was from R&D Systems (Minneapolis, MN). HGF was a kind gift of Genentech (South San Francisco, CA). The MET inhibitor Crizotinib was from Pfizer (New York, NY), the EGFR inhibitor Erlotinib was from Sigma (St. Louis, MO). Human type IV collagen was purchased from Sigma (St Louis, MO). Crystal Violet was purchased from Promega Corp (Madison, WI). Alamar blue was obtained from Invitrogen (Grand Island, NY). All microRNAs were obtained from Ambion-Biosystems (Huston, TX). The miRNeasy kit, miScript Reverse Transcription Kit and human U6B probe, all microRNA Primer Assays and Universal Primers were purchased from Qiagen (Valencia, CA). The EGFR, MET and β-actin antibodies for immunoblotting were purchased from Santa-Cruz Biotechnology (Santa Cruz, CA) and Bcl-xL antibody was purchased from Cell Signaling (Carlsbad, CA). Annexin V-PE, and 7AAD were from BD Pharmingen (San Diego, CA).

### Vectors

The MET 3′-UTR reporter plasmid was a kind gift from Dr. Lin He (Cold Spring Harbor Laboratory, Cold Spring Harbor, NY) [16]. The EGFR 3′-UTR reporter plasmid was a kind gift from Dr. Benjamin Purow (University of Virginia, VA) [17]. It was constructed via insertion of the EGFR 3′-UTR into the *Xba*I restriction site 3′ to luciferase in the pGL3-promoter plasmid (Promega Corp.). The Bcl-xL 3′UTR-Luc plasmid was purchased from GeneCopoeia (Rockville, MD).

### Immunohistochemistry

To assess the expressions of EGFR and MET in chordoma, 4% formalin fixed, paraffin embedded human surgical tumors were subjected to immunohistochemistry using EGFR or MET specific antibodies as previously described [18]. Representative fields were photographed at 40X magnification.

### Quantitative PCR

Small RNAs were isolated from chordoma cells and fibroblasts using the miRNeasy kit according to the manufacturer's instructions (Qiagen). 500 ng of RNA was used to generate cDNA using the miScript reverse transcription kit. From 100 ng of cDNA template, quantitative real-time PCR analyses for 26 miRNAs (miR-335, miR-202, miR-29a, miR-20a*, miR-34a, miR-148a, miR-23b, miR-23b, miR-10b, miR-204, miR-221, miR-297, miR-328, miR-421, miR-663, miR-7, miR-134, miR-196, mIR-582-3p, miR-885-5p, miR-768-5p, miR-582-5p, miR-887, miR-363-miR-22, miR-768-3p, and mir-608) and U6B control were performed using miRNA specific primer assays and U6B-specific primers according to the manufacturer's protocol (Qiagen). U6B was used as a control to normalize the levels of miRNAs tested. Applied Biosystems (StepOnePlus) real-time PCR system was used to perform the quantitative PCR, using hot start, with annealing temperature at 55°C (30 s), extension 70°C (30 s) for 40 cycles, followed by a melt curve analysis. Data analysis was carried out using StepOne software V2.1 (Applied Biosystems).

### MiRNA gene copy number determination

Purified genomic DNA from chordoma cells, fibroblasts and astrocytes was analyzed by Taqman quantitative real-time PCR in triplicates using sets of PCR primers/probes targeting the miR-608 (10q24.31) and miR-34a (1p36.22) on chromosome 10 and 1, and the MCM7 (7q21.3-q22.1) gene on chromosome 7 as a reference control [12]. The primer sequences were: for miR-608: 5′-TCAGGAGTCAGAGTGCAGGA-3′ and 5′-CGGACCAGGATGAGAGAGAG-3′, 608-Probe 5′-[6-FAM]CGGCAGCATTTGTTGGGAGAAACG[Tamra]-3′; for miR-34a: 5′-TCAGGAGTCAGAGTGCAGGA-3′, 5′-CGGACCAGGATGAGAGAGAG-3′, 34a-Probe 5′-[6-FAM]CGGCAGCATTTGTTGGGAGAAACG[Tamra]-3′; for MCM7: 5′-CGTGAGTGGAGAACTGACC-3′, 5′-CAGCCATCTTGTCGAACTC-3′, and MCM7-Probe 5′-[6-FAM]TGACCAGGGTGTGTGCTGCA[Tamra]-3′. Reactions were run according to the Taqman Copy number Assay: 10 ul of 2X TaqMan Genotyping master mix, 1 ul of TaqMan Copy number Assay 20X working stock, 1 ul of TaqMan Copy number Reference Assay, 20 ng of DNA or genomic standards in 20 ul total reaction volume. PCR was performed using the Applied Biosystems 7500 Real-Time PCR System with thermocycling at: 95C 10 minutes, then 95C 15 sec, 60C 60 sec for 40 cycles. Ten non-chordoma blood DNA samples were used to establish a normal reference miRNA:MCM7 ratio. The test samples were normalized to the normal reference ratio and multiplied by 2 to obtain the absolute estimated miRNA copy number. Experimental results were analyzed by CopyCaller V2.0 software provided by Applied Biosystems.

### Pharmaceutical inhibition of EGFR and MET

Chordoma cells were treated with Erlotinib (5 µM) or Crizotinib (300 nM) for 1–5 days and were then assessed for growth with the alamar blue assay. Cells were loaded with alamar Blue reagent at 10% and incubated at 37°C in 5% CO2 for 40–60 minutes. 200 μl of medium was transferred to 96-well plates in triplicates and fluorescence measured at 544 nm/590 nm (Ex/Em) using the Thermao Scientific Varioskan Glash Plate Reader.

### MiRNA transfections

miRNA precursors pre-miR-608, pre-miR-34a and pre-miR-control (pre-miR-con) were used to overexpress miR-608 or miR-34a in chordoma and normal cells. Cells were plated at 50% confluence and transfected with pre-miR-608 and pre-miR-34a using oligofectamine transfection reagent. Pre-miR-control (pre-miR-con) was used as a control. Overexpression of miR-608 or miR-34a was verified by quantitative RT-PCR.

### Immunoblotting

Immunoblotting was performed using antibodies specific for EGFR, Bcl-xL, MET and β-Actin as a loading control. Chordoma cell line UCH1 and primary cells C24 were transfected with pre-miR-608 or miR-34a or pre-miR-con for 48 hours, lysed in RIPA buffer, and immunoblotted for the different predicted target proteins of miR-608 or miR-34a as previously descripted [19]. All blots were stripped and re-probed with β-Actin antibody as loading control.

### Proliferation assay

To assess the effect of miR-608 and miR-34a overexpression on cell growth, 20, 000 chordoma cells were seeded in triplicates in medium containing 10% FBS, and transfected either with pre-miR-608, pre-miR-34a or pre-miR-con (20 nM). 72 hours later, the cells were harvested every day for 5 days or every other day for 9 days (depending on observed growth rates) and counted with a hemocytometer and growth curves were established.

### Annexin V-PE and 7AAD flow cytometry

Cell death and apoptosis were assessed by Annexin V-PE and 7AAD flow cytometry as previously described [20]. Briefly, cells were transfected with pre-miR-608, pre-miR-34a or pre-miR-con for 96 hrs. The cells were harvested and stained with Annexin V-PE and 7AAD according to the instructions of the manufacturer. Cell samples were analyzed on a FACsan and apoptotic and dead cell fractions were determined.

### Invasion assay

Chordoma cells were treated with 20 nM pre-miR-608, pre-miR-34a or pre-miR-control for 72 hours. The cells (2.5×10^5^) were seeded in low serum medium (0.1%) on collagen IV-coated inserts. 600 μl of complete medium was placed in the lower chamber as a chemoattractant. After 8–16 hours of incubation at 37°C, invading cells were stained with 0.1% crystal violet solution and photographed at 40X. The cells were then counted under the microscope in five randomly chosen fields and the number of invading cells was used for comparative analyses by one-way ANOVA.

#### 3′-UTR reporter assays

3′UTR luciferase assays were used to determine if miR-608 directly binds to the EGFR and Bcl-xL 3′UTR and if miR-34a directly binds to the MET 3′UTR. Cells were transfected with pre-miR-608, pre-miR-34a or pre-miR-con for 6 hrs. The cells were then transfected with either 3′UTR control, 3′UTR-EGFR, 3′UTR-Bcl-xL or 3′UTR-MET, in addition to control cytomegalovirus-β-galactosidase (β-Gal) reporter plasmids for 48 hrs. Luciferase assays were performed using the Luciferase System Kit (Promega, Madison, WI) and luminescence was measured. Firefly luciferase activity was double normalized by dividing each well first by β-galactosidase activity and then by average luciferase/β-galactosidase in a parallel set with a constitutive luciferase plasmid.

### Statistics

All experiments were performed at least in triplicates. Numerical data are expressed as mean ± standard deviation. Two group comparisons were analyzed by two-sided Student's t test. The statistical associations between miRNA and protein expression were evaluated with regression correlation analyses. The correlation coefficient “R” was calculated. P values were determined for all analyses and p<0.05 was considered significant and symbolized by an asterisk in the figures.

## Results

### MiRNAs are differentially expressed in chordoma

**Figure 1:**
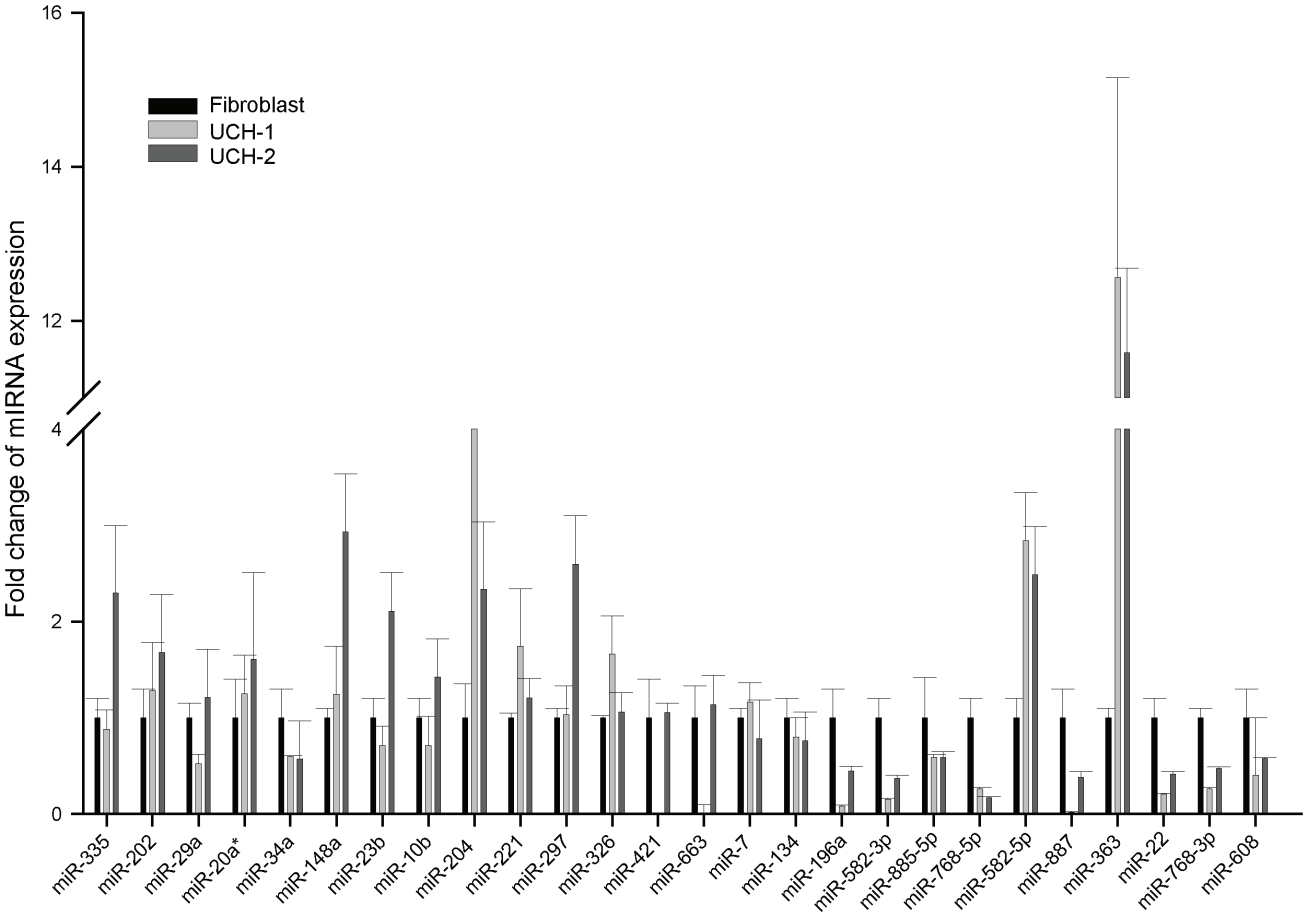
microRNAs are differentially expressed in chordoma cells. Small RNAs were extracted from chordoma UCH1 and UCH2 cells and control fibroblasts. miRNA levels were measured using qRT-PCR relative to control U6B snRNA.

miRNAs have been studied extensively in cancer and found to play important roles in regulating gene expression and malignancy. However, very limited information about miRNAs in chordoma is available. To assess the roles of miRNAs in chordoma, we measured the expressions of 26 miRNAs in two chordoma cell lines (UCH1 and UCH2) as compared to normal human fibroblasts and astrocytes using qRT-PCR. We chose fibroblasts and astrocytes as normal controls because the extreme difficulty in obtaining normal notochord cells. To our knowledge, worldwide only one lab (Congenital Defects’ Lab at the University of Washington) has very limited amounts of human notochordal tissue and there are no notochord cell lines known. The miRNAs chosen in the screening were based on data from our studies in glioblastomas which demonstrated their importance in this tumor type. We found significant and consistent differential expression of several miRNAs that mostly but not completely mirrored the expression changes observed in glioblastomas (Figure 1 and Figure S1). The differentially expressed miRNAs relative to fibroblasts included the strongly upregulated miR-363 (∼12 fold up), miR-204 (∼ 5 fold up), miR-582-5p (∼ 3 fold up) and the significantly downregulated miR-608 (∼70% down), miR-34a (∼60% down), miR-768-5p (∼80% down), and miR-582-3p (∼70% down). (Figure 1). We also compared the miRNA levels in chordoma cell lines to astrocytes and found the similar pattern as to fibroblasts (figure S1). Among other, we observed that miR-1 is down regulated in both chordoma cell lines, which is consistent with a previously published study (figure S1). This shows that miRNAs are deregulated in chordoma where they might play a role in gene expression regulation and malignancy. We selected two miRNAs for further study based on their predicted targeting of genes known to play roles in chordoma malignancy. These were miR-608, which has potential binding sites in the 3′UTR of EGFR and Bcl-xL mRNAs (which are overexpressed in almost all chordoma tumors) and miR-34a which is predicted to target MET (expressed in up to 100% of chordomas) [21]–[23].

### EGFR and MET are expressed in chordoma where they promote cell proliferation and invasion

**Figure 2:**
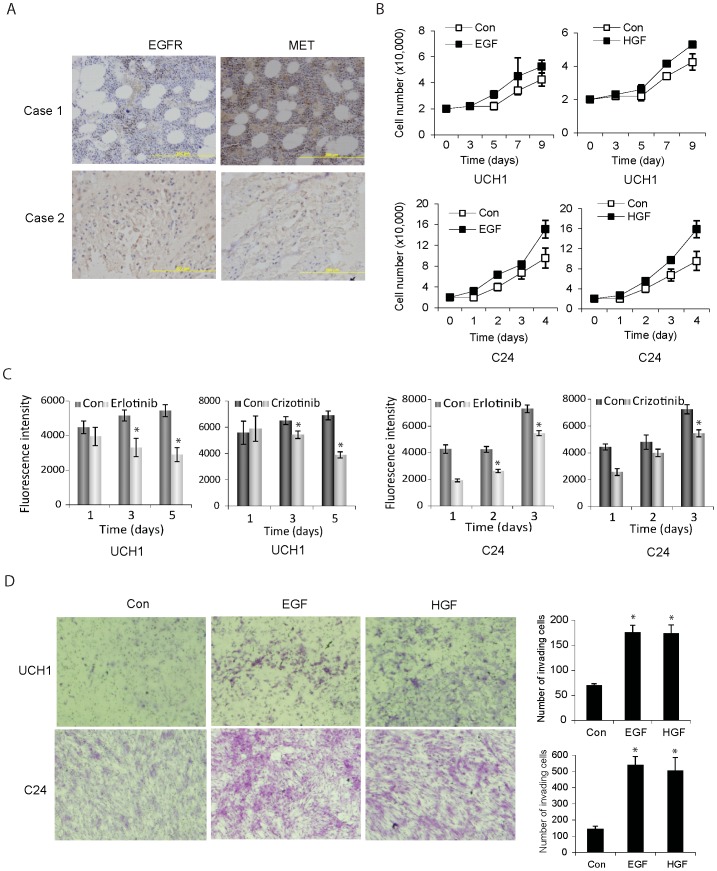
EGFR and MET are highly expressed in human chordoma specimens and activation of EGFR and MET enhances chordoma cell proliferation and invasion. A) Immunohistochemical staining in two representative formalin-fixed, paraffin-embedded chordoma tumors. Strong EGFR and MET staining are observed. B) Chordoma cell line UCH1 and primary cells C24 were treated with EGF or HGF every 24 hrs and then assessed for proliferation by cell counting. The results show that EGF and HGF enhance chordoma cell proliferation. C) Chordoma cells UCH1 and C24 were treated with Erlotinib or Crizotinib for 1–5 days and then assessed for cell growth by alamar blue assay. The data indicate that Erlotinib and crizotinib inhibit chordoma cell growth. D) UCH1 and C24 cells were treated with EGF and HGF for 24 hrs and subsequently assessed for cell invasion using a transwell assay. The data show that EGF and HGF strongly promote cell invasion through the transwell.

EGFR and MET are among very few molecules that have been shown to be deregulated in human chordoma. It was shown that 67–100% of chordomas overexpress EGFR and 70–100% overexpress MET (Table 1). However, overexpression of these molecules can be only partly explained by gene amplification. In fact, studies have found that 27–40% of chordomas have amplified EGFR and 27–50% have amlplified MET genes (Table 1). To confirm the expression of EGFR and MET in chordoma specimens, we performed immunohistochemistry on chordoma tumor sections. We found high expressions of EGFR and MET in chordoma specimens (Figure 2A). We then determined the effects of EGFR and MET activations on chordoma malignancy endpoints. We activated EGFR with EGF (20 ng/ml) or MET with HGF (20 ng/ml) treatment in UCH1 and C24 chordoma cells. We then assessed them for proliferation by cell counting and invasion by transwell invasion through a membrane coated with collagen IV, a commonly expressed matrix protein in chordoma. We also inhibited EGFR and MET activity with Erlotinib and Crizotinib, respectively, and measured cell growth by alamar blue assay. We found that EGFR and MET activations enhanced cell proliferation (Figure 2B), and promoted invasion in both UCH1 and C24 cells (Figure 2D), while inhibition of EGFR and MET inhibited cell growth (Figure 2C). The above data suggest that EGFR and MET play important roles in chordoma formation and regulate chordoma cell proliferation and invasion.

**Table 1:** EGFR and MET expression and gene amplification in chordoma in published immunohistochemical studies.

	Chromosome	Number of tumors	Overexpression	Chromosome bisomy	References
EGFR	7p11.2	12–173	67–100%	27–40%	3, 21, 39, 41
MET	7q31	12–66	70–100%	27–50%	21, 22, 23

### MiR-608 is downregulated and inversely correlates with EGFR levels in chordoma cells

**Figure 3:**
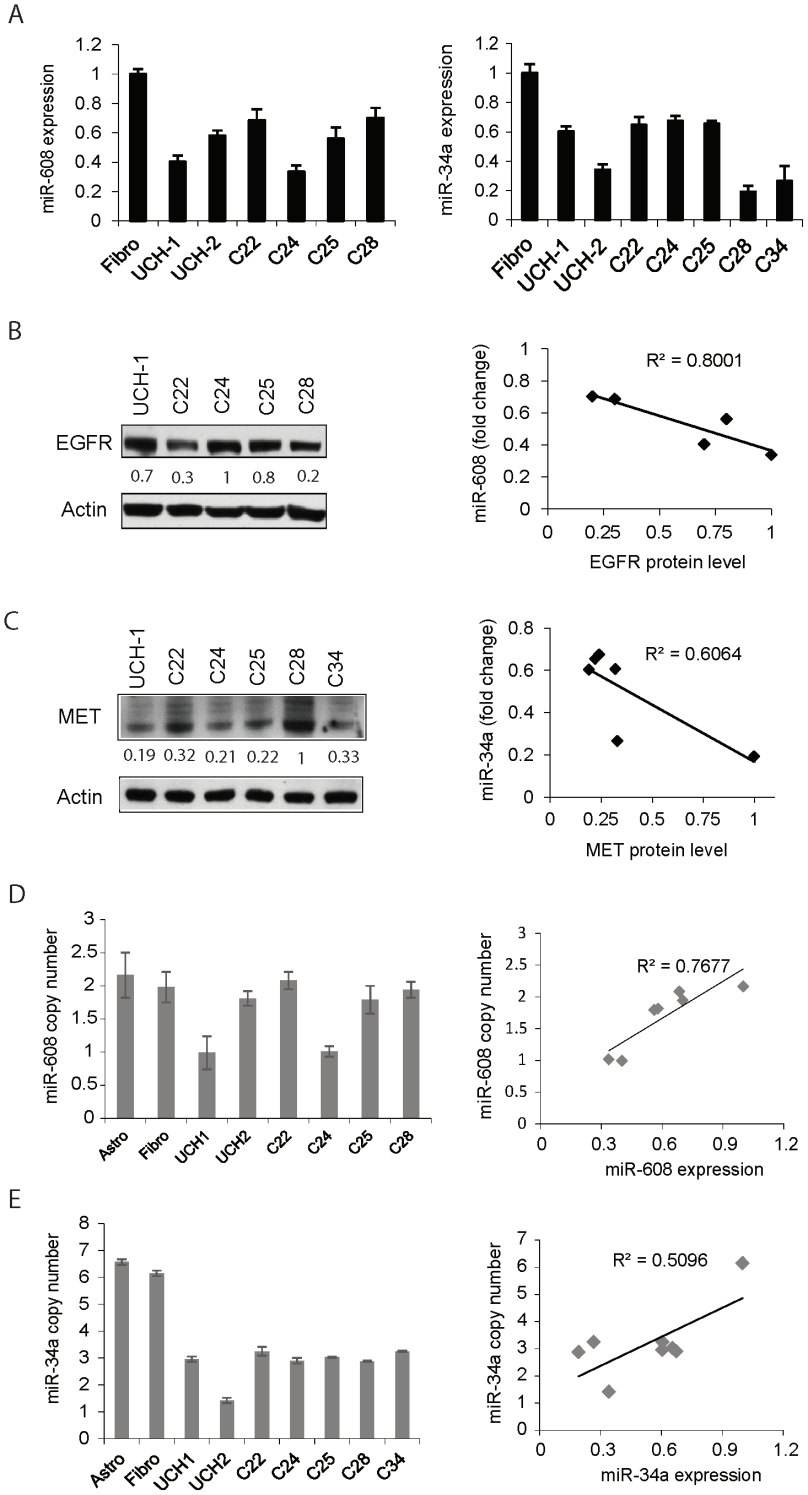
miR-608 and miR-34a are downregulated via gene copy number alteration, and miR-608 and miR-34a expressions inversely correlate with EGFR and MET in chordoma cells. A) miR-608 and miR-34a levels in chordoma cell lines and primary cells were measured by qRT-PCR and compared with those in fibroblasts, B) lystaes from chordoma cells were immunoblotted for EGFR and normalized to β-actin (left panel), and the level of miR-608 was correlated with EGFR protein level (right panel) (R^2^ = 0.8, P<0.05). C) MET protein levels in chordoma cells were determined by immunoblotting (left panel) and miR-34a levels correlated with MET protein (right panel) (R^2^ = 0.0.61, P<0.05). D) miR-608 gene copy numbers in chordoma cells were determined by q-PCR (left panel) and miR-608 expression levels were correlated with gene copy number (R^2^ = 0.77, P<0.05) (right panel). E) miR-34a gene copy numbers in chordoma cells were determined by q-PCR (left panel) and miR-34a expression levels are correlated with gene copy number (R^2^ = 0.51, P<0.05).

We measured miR-608 levels by qRT-PCR and EGFR protein levels by immunoblotting in chordoma cell lines, primary cells and normal fibroblasts. We found that median miR-608 levels are ∼ 50% lower than in normal fibroblasts (Figure 3A, left panel). We analyzed the correlation of miR-608 and EGFR expressions in chordoma cells and found that miR-608 level is inversely correlated with EGFR protein levels (Figure 3B) (R^2^ = 0.8, P<0.05). These data show that miR-608 is downregulated in chordoma and suggest that it is an important regulator of EGFR expression.

### MiR-34a is downregulated and inversely correlates with MET expression in chordoma cells

We measured miR-34a levels by qRT-PCR and MET protein levels by immunoblotting in chordoma cell lines, primary cells and normal fibroblasts. We found that median miR-34a levels are ∼ 60% lower in chordoma cells than in normal fibroblasts (Figure 3A, right panel). We analyzed the correlation of miR-34a and MET expressions in chordoma cells and found that miR-34a level inversely correlated with MET protein levels (Figure 3C) (R^2^ = 0.61, P<0.05). These data show that miR-34a is downregulated in chordoma and suggest that it is an important regulator of MET expression.

### MiR-608 and miR-34a loci display gene copy number losses that correlate with downregulated miRNA in chordoma

The loci of miR-608 in chromosome 10 and miR-34a in chromosome 1 have been reported to be deleted in chordoma [24]. We therefore determined gene copy numbers for both miRNAs in chodoma cell lines and primary cells. We performed quantitative PCR in chordoma cells using one set of primer/probes targeted to miRNAs and a reference control primer/probe set targeting MCM7 and RNaseP. Chordoma cells UCH1 and C24 showed a 50% decrease of miR-608 copy number as compared to fibroblasts or astrocytes (Figure 3D, left panel). miR-34a copy number in all chordoma cells was about 47–76% less than in fibroblasts or astrocytes (Figure 3E, left panel). We then analyzed the correlation between the copy number and miRNA expression. We found a significate linear correlation between mature miR-608 levels and miR-608 gene copy number in chordoma cells (R^2^ =  0.77, P<0.05) (Figure 3D, right panel). We also found that miR-34a expression correlates with miR-34a gene copy number (R^2^ =  0.51, P<0.05) (Figure 3E, right panel). These data suggest that miR-608 and miR-34a downregulation in chordoma cells is (at least partially) due to gene copy number decrease.

### Identification of EGFR and Bcl-xL as targets of miR-608, and MET as a target of miR-34a in chordoma cells

**Figure 4:**
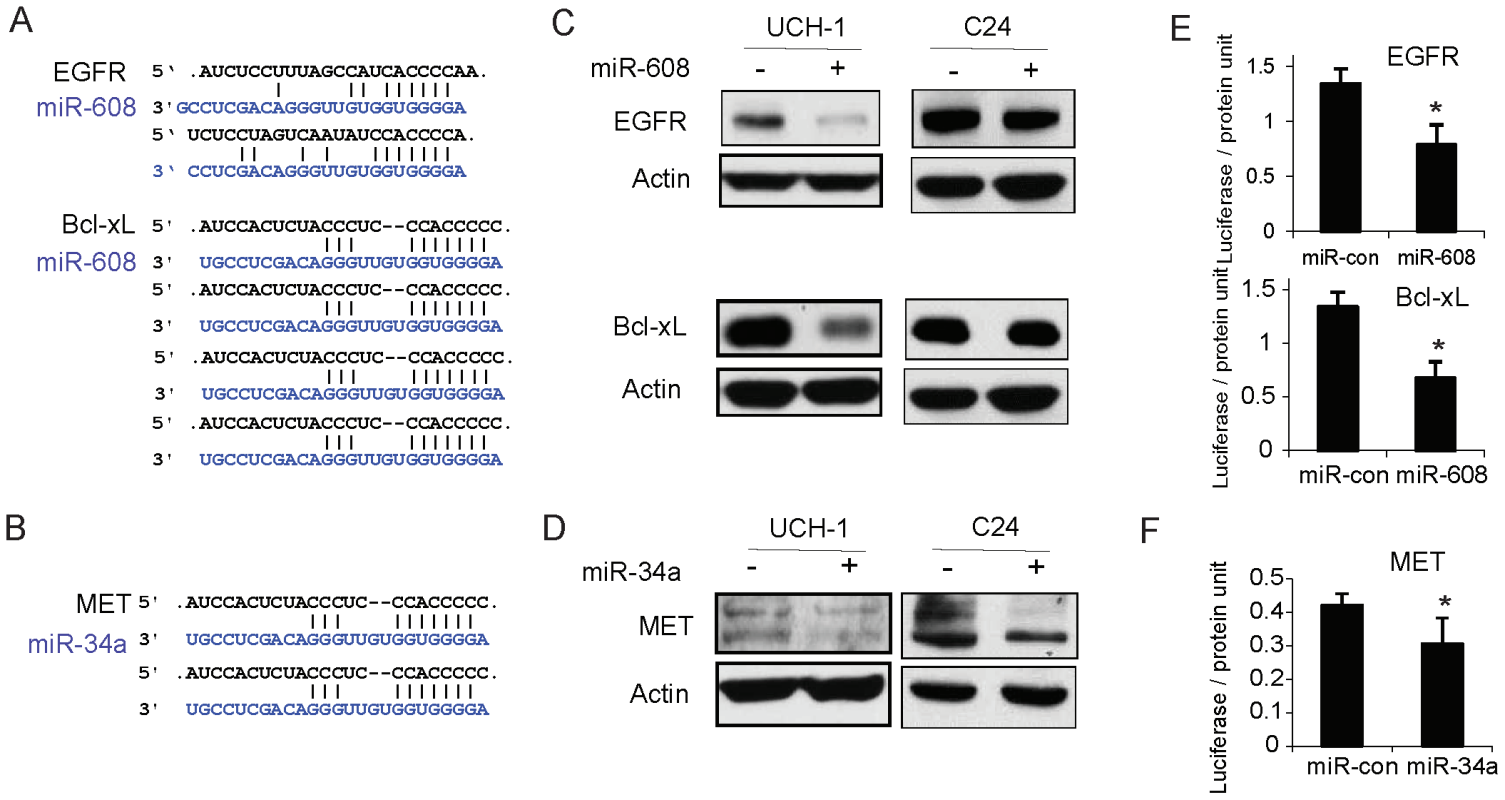
miR-608 downregulates EGFR and Bcl-xL by directly binding to their mRNA 3′UTR and miR-34a downregulates MET by directly binding MET 3′UTR. A) Predicted binding sequences of miR-608 in the 3′UTR sequences of EGFR and Bcl-xL mRNA; B) Predicted binding sequences of miR-34a in the 3′UTR sequence of MET mRNA; C), D) UCH1 and C24 cells were transfected with pre-miR-608 (C) or pre-miR-34a (D) or control pre-miR for 48 hrs. Cell lysates were immunoblotted for EGFR or Bcl-xL (C) or MET (D), The results show that the miRNAs significantly inhibited these predicted target proteins in chordoma cells; E), F) UCH1 cells were transfected with pre-miR-608, pre-miR-34a or pre-miR-con and then with either EGFR 3′UTR, Bcl-xL 3′UTR, MET 3′UTR or control reporter plasmids together with β-Galactosidase (β-Gal) plasmid, and 3′UTR reporter activity was measured by a luciferase assay and normalized to β-Gal activity. The results show that miR-608 expression down-regulates EGFR and Bcl-xL luciferase activities (E) and that miR-34a expression repressed MET luciferase activity (F) in UCH1 cells. (* P<0.05)

We used miRNA target prediction analysis (Figure 4A, 4B), immunoblotting and 3′-UTR reporter assays to search for oncogenes that are directly targeted by miR-608 or miR-34a in chordoma cells. We selected proteins that are known to play roles in chordoma and other cancers. We transfected pre-miR-608, pre-miR-34a or pre-miR-control (pre-miR-con) in UCH1 and C24 cells and verified transfection levels, which were 3-6 fold higher than in control (not shown). miR-608 transfection downregulated EGFR and Bcl-xL proteins and miR-34a downregulated MET protein as assessed by immunoblotting (Figure 4C, 4D). To determine if miR-608 and miR-34a directly inhibit EGFR, Bcl-xL or MET protein expressions by binding to their mRNA 3′-UTR, we performed 3′UTR reporter luciferase assays. miR-608 transfection significantly inhibited EGFR and Bcl-xL 3′-UTR luciferase activities in UCH1 cells (n = 3; P<0.05) (Figure 4E). miR-34a transfection significantly inhibited MET 3′-UTR luciferase activity (Figure 4F) (n = 3; P<0.05). These data show that miR-608 and miR-34a respectively regulate EGFR, Bcl-xL and MET by acting on their 3′UTRs.

### MiR-608 and miR-34a induce chordoma cell apoptosis

**Figure 5:**
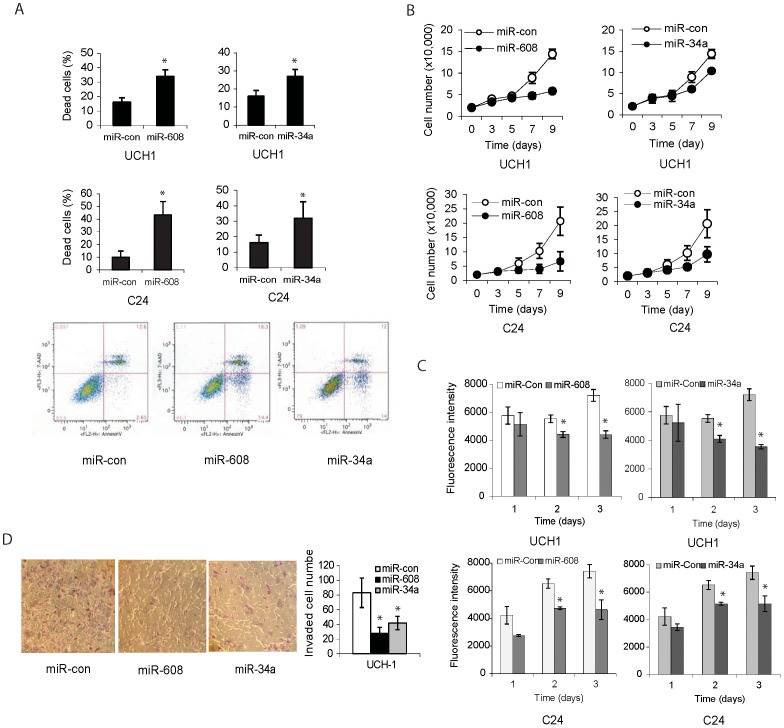
miR-608 and miR-34a inhibit chordoma cell proliferation, survival and invasion. **A)** UCH1 chordoma cell line and C24 primary cells were transfected with pre-miR-608 or pre-miR-34a (20 nM) for 96 hrs and subsequently assessed for apoptosis using AnnexinV-PE/7AAD flow cytometry. The results show that miR-608 (upper panel) and miR-34a (middle panel) significantly induce cell apoptosis. The bottom panel shows representative flow cytometry data; (* P<0.05). **B), C)** UCH1 and C24 cells were transfected with pre-miR-608 or pre-miR-34a for 72 hrs and then assessed for proliferation by cell counting (B) or for cell growth by alamar blue assay (C). The data show that miR-608 (left panel) and miR-34a (right panel) inhibit the proliferation and growth of chordoma cells. **D)** UCH1 cells were transfected with pre-miR-608 or pre-miR-34a for 72 hrs, and subsequently assessed for cell invasion using a transwell assay. Cells that invaded through the membrane after 16 hrs were fixed, stained and counted. The results show that miR-608 and miR-34a strongly reduced the ability of cell invasion through the transwell (left panel). Quantification of these latter data is shown in the right panel. (* P<0.01)

One of the hallmarks of chordoma and other cancers is the loss of programmed cell death. To determine if miR-608 or miR-34a plays a role in regulating chordoma cell apoptosis, we transfected pre-miR-608 or pre-miR-34a in UCH1 chordoma cell line and C24 primary cell and measured apoptosis with Annexin V/7AAD flow cytometry. We found that miR-608 or miR-34a strongly induced cell apoptosis (Figure 5A).

### MiR-608 and miR-34a inhibit chordoma cell proliferation and growth

To determine the effects of miR-608 and miR-34a on cell proliferation, we transfected UCH1 and C24 chordoma cells with pre-miR-608 or pre-miR-34a and assessed cell proliferation by cell counting. We found that miR-608 and miR-34a significantly inhibited chordoma cell proliferation (Figure 5B). We also assessed cell growth by the alamar blue assay and found that miR-608 and mIR-34a significantly inhibited chordoma cell growth (Figure 5C).

### MiR-608 and miR-34a inhibit chordoma cell invasion

Chordoma malignancy is in part due to its invasive growth that affects important functions of the surrounding central or peripheral nervous systems and causes distant metastasis to lung, liver, bone, and skin. To determine if downregulation of miR-608 or miR-34a is responsible for chordoma cell invasive growth, we studied the effects of miR-608 and miR-34a on chordoma transwell invasion using a transwell assay. We found miR-608 and miR-34a strongly reduced UCH1 cell invasion through a membrane coated with collagen IV (Figure 5D). miR-608 reduced invasion by ∼ 75% and miR-34a reduced it by ∼ 50% (Figure 5D, right panel). Therefore, miR-608 and miR-34a might act as tumor suppressive molecules in chordoma cell by inhhibiting cell invasion.

## Discussion

Our study uncovers several deregulated miRNAs in chordoma including miR-608 and miR-34a. Among the list of miRNAs tested, we also found several other dramatically deregulated miRNAs, which are known to play important roles in other cancer types. For example, miR-196a has been studied for its oncogenic roles in glioblastoma, pancreatic adenocarcinoma, breast cancer, oesophageal adenocarcinoma, and colorectal cancer, and shown to target HOX family genes (HOXA7, HoxB7, HOXC8, HOXB8, HOXD8), ERG, HMGA2, ANXA1, S100A9, SPRR2C, KRTS [25] [26]–[31]. miR-196a associates with worse survival in glioblastoma and pancreatic adenocarcinoma [31], [32]. miR-22 has been reported to play an important role in tumorigenesis in a cell-type specific manner. The expression of miR-22 is increased in human senescent fibroblasts and epithelial cells and targets PTEN, p21, and p53, but it is decreased in a variety of cancer cells such as colon cancer, liver cancer, ovarian cancer, and breast cancer cells [33]–[36]. miR-885-5p is upregulated in head and neck cancer and oncocytic follicular carcinomas and acts by targeting caspase 3 [37]. miR-885-5p also targets CDK2 and MCM5, activates p53 and inhibits proliferation and survival in neuroblastoma [38].

We chose miR-608 and miR-34a for further study because their chromosomal loci are frequently deleted in chordoma [24] and because they are predicted to target EGFR and MET, which are the most commonly deregulated RTKs in chordoma [4], [39], [40]. Our data show that miR-608 and miR-34a expression levels are much lower in chordoma cell lines and primary cells than in normal human fibroblasts and astrocytes. miR-608 and miR-34a expressions negatively correlated with the most commonly overexpressed oncogenes EGFR/Bcl-xL and MET, respectively. Restoration of miR-608 or miR-34a repressed cell proliferation, induced apoptosis, and decreased cell invasion in chordoma cell lines and primary cells. Pharmaceutical inhibition of EGFR or MET showed similar effects on cell cell growth as restoration of mIR-608 or miR-34a. We identified EGFR and Bcl-xL as direct targets of miR-608. We also identified MET as a direct target of miR-34a. We therefore identified and characterized miR-608 and miR-34a as novel tumor suppressive miRNAs in chordoma that directly target EGFR, Bcl-xL or MET.

Chordomas are rare but very malignant tumors. Very little is known about the underlying molecular mechanisms and the role of miRNAs in chordomas. Most published work on chordoma consists of descriptive pathological and genetic studies. Few molecular and functional studies have been published. This is in part due to the very limited investigative tools for chordoma research. In fact, there are currently only two chordoma cell lines available (the ones used in this study) and no well-established animal model.

A few studies have shown that EGFR is one of the most frequently activated RTKs in chordomas [4], [39], [40] and that its activation correlates with aggressive tumor behavior [21]. This suggests that EGFR activation is an important driver of chordoma malignancy. EGFR is overexpressed in up to 100% of chordoma tumors, but is amplified in up to 40% of tumors [3], [21], [39], [41] (Table 1). Therefore, EGFR gene amplification only partially accounts for EGFR overexpression. This implies the existence of mechanisms other than gene amplification that lead to EGFR overexpression. Our study uncovers for the first time miR-608 downregulation as a new mechanism of EGFR overexpression in chordoma.

We also identified the anti-apoptotic molecule Bcl-xL as a target of miR-608. Bcl-xL is a member of the Bcl-2 family that acts as an anti-apoptotic protein by preventing the release of mitochondrial contents that lead to caspase activation [42], [43]. It has been shown that Bcl-xL overexpression contributes to genetic instability, resulting in increased tetraploid cells [44]. It has been reported that Bcl-xL is regulated by EGFR-activated STAT3 to promote survival in head and neck cancer [45]. Another study demonstrated that nuclear EGFRvIII-STAT5b complex directly activates the Bcl-xL promoter and contributes to cell survival in glioblastoma [46]. This strongly suggests that combined inhibition of Bcl-xL and EGFR is likely to achieve synergistic anti-tumor effects. Our study shows that such combined targeting of Bcl-xL and EGFR can be achieved by overexpression of miR-608, providing a novel and powerful rationale for the therapeutic restoration of this microRNA in chordoma therapy.

The receptor tyrosine kinase MET and its ligand hepatocyte growth factor (HGF) are expressed in a variety of solid tumors, including chordomas and have emerged as key determinants of tumor growth and invasion [47]–[49]. Overexpression of MET and HGF in chordoma enhances cell survival and invasion [47], [49]. MET is overexpressed in up to 100% of chordoma as determined by a study of 66 tumor samples among which about 50% showed gene amplification [21]–[23] (Table 1). Therefore about 50% MET overexpression is caused by mechanisms other than gene amplification. Our study shows that downregulation of miR-34a is one such mechanism for MET overexpression.

Our data show that the gene copy number of miR-608 and miR-34a is reduced in a majority of chordoma cells and that copy number is correlated with the downregulated expression levels of miR-608 and miR-34a. Therefore, copy number alterations of miR-34a and miR-608 are likely partly responsible for EGFR, MET and Bcl-xL overexpressions in chordoma as demonstrated by our study. This suggests that miR-608 and/or miR-34a restoration could be a novel therapeutic strategy for chordoma. Because microRNAs are naturally existing small molecules and target multiple genes, overexpression of miR-608 or miR-34a could exert greater anti-tumor effects than EGFR or MET inhibitions.

In summary, our study shows for the first time that miR-608 and miR-34a are deregulated tumor suppressive miRNAs that act via regulation of EGFR, MET and apoptosis in chordoma.

## Supporting Information

Figure S1 **microRNAs are differentially expressed in chordoma cells.** Small RNAs were extracted from chordoma UCH1 and UCH2 cells and control astrocytes. miRNA levels were measured using qRT-PCR relative to control U6B snRNA.
(TIF)
