# Supplementary figures and images for: MicroRNA-608 and MicroRNA-34a Regulate Chordoma Malignancy by Targeting EGFR, Bcl-xL and MET
Source: PLoS One. 2014 Mar 12;9(3):e91546. doi: 10.1371/journal.pone.0091546 (PMC3951453; doi:10.1371/journal.pone.0091546)

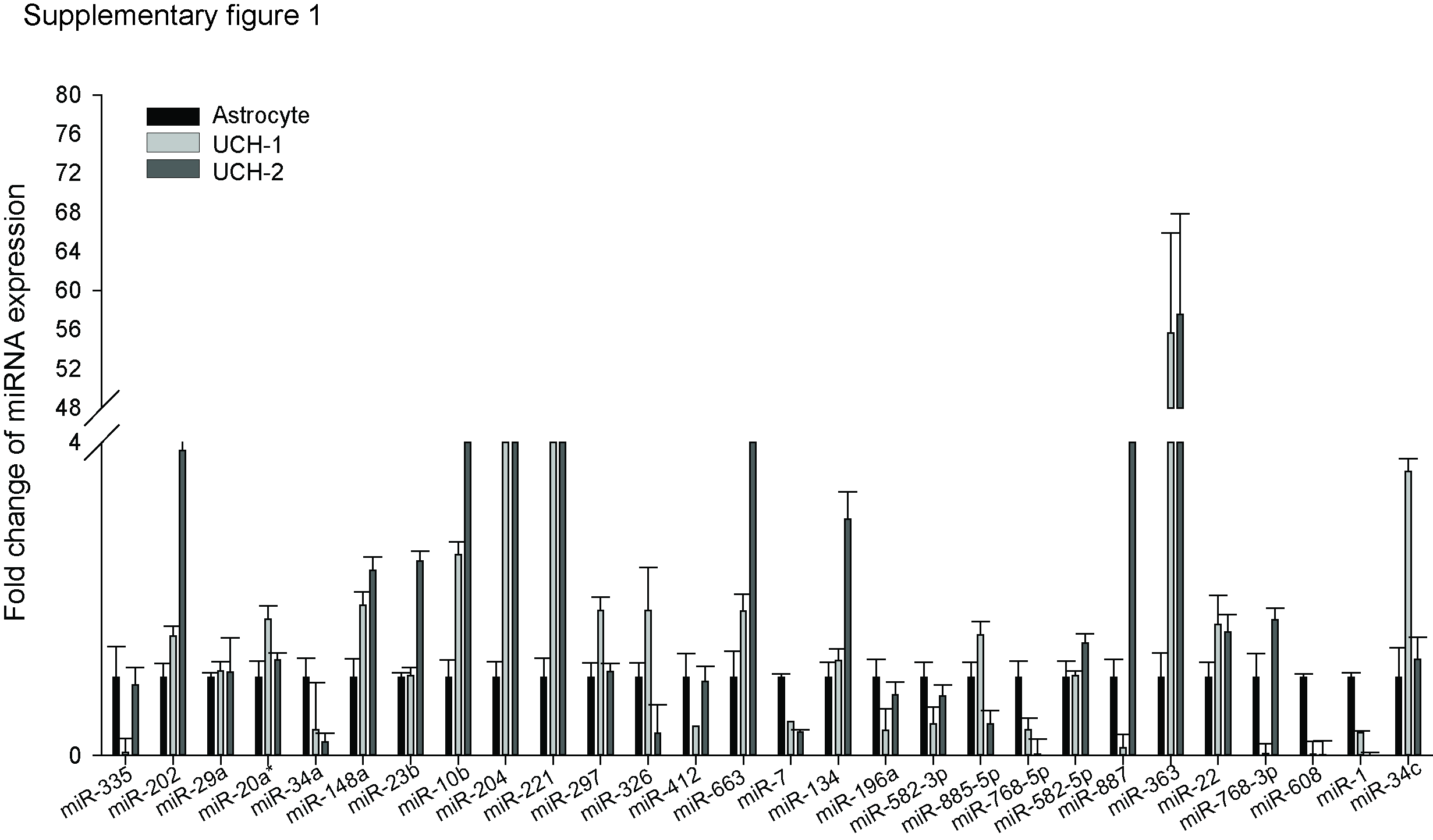

Supplement: Figure S1 — microRNAs are differentially expressed in chordoma cells. Small RNAs were extracted from chordoma UCH1 and UCH2 cells and control astrocytes. miRNA levels were measured using qRT-PCR relative to control U6B snRNA. (TIF) [file pone.0091546.s001.tif]
